# Potential Roles of Exogenous Proteases and Lipases as Prebiotics

**DOI:** 10.3390/nu17050924

**Published:** 2025-03-06

**Authors:** Yongshou Yang, Thanutchaporn Kumrungsee, Yukako Okazaki, Toshiro Watanabe, Junji Inoue, Takafumi Iguchi, Shinji Fukuda, Manabu Kuroda, Kyoichi Nishio, Shotaro Yamaguchi, Norihisa Kato

**Affiliations:** 1 School of Life Sciences, Anhui University, Hefei 230036, China; 21193@ahu.edu.cn; 2 Graduate School of Integrated Sciences for Life, Hiroshima University, Higashi-Hiroshima 739-8528, Japan; 3 Faculty of Human Life Sciences, Fuji Women’s University, Ishikari 061-3204, Japan; yokazaki@fujijoshi.ac.jp; 4 Faculty of Human Health, Sonoda Women’s University, Amagasaki 661-0012, Japan; watnb-ts@sonoda-u.ac.jp; 5 Ahjikan Co., Ltd., Shoko Center, Hiroshima 733-0833, Japan; j-inoue@ahjikan.co.jp; 6 R & D Division Yaegaki Biotechnology, Inc., Himeji 679-4298, Japan; takafumi.iguchi@yaegaki.com; 7 Institute for Advanced Biosciences, Keio University, Tsuruoka 997-0052, Japan; sfukuda@sfc.keio.ac.jp; 8 Transborder Medical Research Center, Faculty of Medicine, University of Tsukuba, Tsukuba 305-8575, Japan; 9 Gut Environmental Design Group, Kanagawa Institute of Industrial Science and Technology, Kawasaki 210-0821, Japan; 10 Amano Enzyme Inc., Nagoya 460-8630, Japan; manabu_kuroda@amano-enzyme.com (M.K.); kyoichi_nishio@amano-enzyme.com (K.N.); shotaro_yamaguchi@amano-enzyme.com (S.Y.)

**Keywords:** fermented foods, protease, lipase, probiotics, prebiotics, intestine, pancreas, microflora, short-chain fatty acid, inflammation

## Abstract

Digestive enzymes, such as proteases and lipases, are widely recognized for their crucial roles in the ripening and production of fermented foods. Digestive enzymes are also used as supplements in nonruminant livestock to enhance feed digestion and promote animal growth. However, information on the effects of exogenous digestive enzymes on gut health and disease remains limited. Notably, recent studies show that consuming proteases and lipases can increase the levels of beneficial bacteria and short-chain fatty acids in rodent gut. These findings led us to hypothesize that intestinal proteases and lipases play beneficial roles by enriching beneficial bacteria. To examine this hypothesis, we reviewed recent studies on the potential effects of exogenous digestive enzymes on gut microbiota composition and overall health. Consistent with the hypothesis, all 13 studies in this review reported significant improvements in animal gut microbiota composition with the dietary supplementation of proteases and lipases. Additionally, the possible mechanisms of the prebiotic-like effects of the enzymes through increased nutrient digestion were discussed. This review explores how exogenous proteases and lipases influence gut microbiota composition and overall health. This is the first review to provide insights into the potential roles of exogenous digestive enzymes as prebiotics.

## 1. Introduction

Digestive enzymes, such as proteases and lipases, are crucial for the ripening and production of fermented foods [1]. Their action in the fermentation process enhances the digestibility of these foods. Digestive enzymes are also gaining attention in nonruminant animal husbandry, where they are used as supplements to enhance feed utilization and promote animal growth [2]. However, information on their health benefits has remained limited. Understanding the effects of exogenous digestive enzymes could provide valuable insights into the health benefits of fermented foods and digestive enzyme supplements. A wide range of digestive enzyme formulations for humans and livestock are currently available, varying in terms of their enzyme type and source (originating from animals, plants, or microorganisms) [3]. The global market for digestive enzyme supplements was valued at USD 699.4 million in 2021 and is projected to reach USD 1.64 billion by 2031 [4].

**Figure 1:**
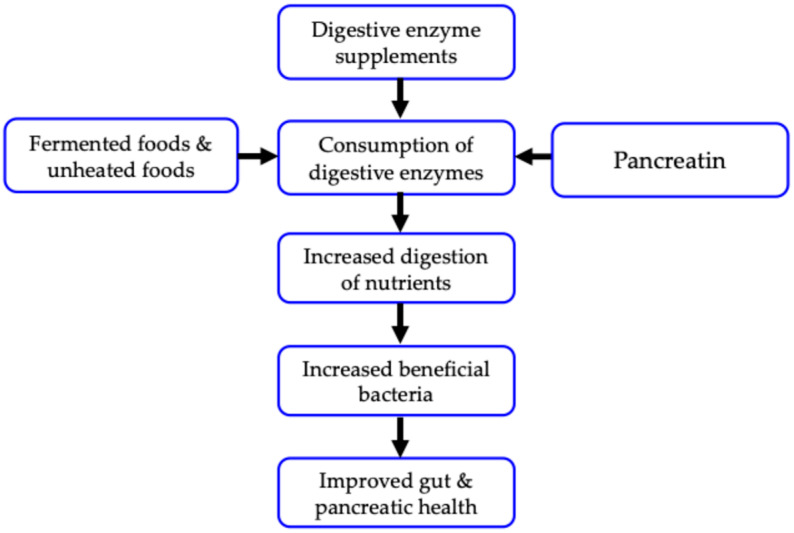
Potential roles of exogenous digestive enzymes as prebiotics.

In 2015, Yang et al. reported that supplementation with *Aspergillus*-derived protease substantially increased the cecal abundance of beneficial bacteria, such as *Bifidobacterium* and *Lactobacillus*, in rats fed a high-fat diet [5]. In 2020, Menden et al. demonstrated that *Candida rugosa* lipase (CL) supplementation in mice significantly increased the fecal levels of beneficial bacteria, including Christensenellaceae and *Akkermansia muciniphila* [6]. These findings led us to hypothesize that supplemental digestive enzymes positively influence the gut microbiome, improving gut health (Figure 1). In 1985, Edward Howell proposed a similar perspective on the benefits of exogenous digestive enzymes in his book “Enzyme Nutrition: The Food Enzyme Concept”, published by Lotus Press [7]. However, his view did not account for the role of the gut microbiota. To date, no reviews have been published on the impact of exogenous digestive enzymes on the gut microbiota [8,9].

In this review, we aimed to examine this hypothesis (Figure 1) by reviewing the relevant literature from databases, such as PubMed, Scopus, and Google Scholar, published from 1980 onward, using the following keywords: “protease”, “lipase”, “intestine”, “microflora”, “fermentation”, “probiotics”, “prebiotics”, pancreas”, and “short-chain fatty acids”. The date of the final electric search was 8 December 2024. The titles and abstracts of the retrieved articles were screened to select potentially relevant articles. Full texts were analyzed to determine whether they were eligible for inclusion. The references in these articles were manually searched to identify further relevant articles. Articles written in languages other than English were excluded. To our knowledge, this is the first review consolidating the current knowledge on the potential effects of exogenous proteases and lipases on the gut microbiota and overall health (Figure 1).

## 2. Proteases

### 2.1. Effects of Supplemental Proteases on the Gut Microbiota

All nine studies [5,10,11,12,13,14,15,16,17] listed in Table 1 demonstrated that feeding animals with protease supplementation significantly improved their gut microbiota composition. Yang et al. [5] first reported that the consumption of *Aspergillus*-derived protease preparations, such as Amano protease (AP) or Orientase (OR), markedly increased the relative abundances of *Bifidobacterium* and *Lactobacillus* and short-chain fatty acid (SCFA) levels in rats fed a high-fat diet. These changes in *Bifidobacterium*, *Lactobacillus*, and SCFA levels were comparable to those induced by typical prebiotics, such as inulin and fructo-oligosaccharide (FOS) [18]. Yang et al. found that among the different constituents of AP—including alkaline, neutral, and acid protease (AcP)—supplemental purified AcP had a significant bifidogenic effect [10]. In their study, the animals fed on a 0.1% AP-supplemented diet had no diarrhea-like symptoms [10].

Kostiuchenko et al. [13] showed that supplemental bromelain from pineapple and papain from papaya reduced the abundance of pathogenic Proteobacteria and increased the abundance of beneficial *A. muciniphila* in mice. Similarly, Peng et al. [14,15] demonstrated that proteases supplements derived from *Bacillus licheniformis*, *Bacillus subtilis*, and *Aspergillus niger* altered the gut microbiota composition of pigs, specifically increasing SCFA-producing bacterial populations. Yi et al. [16] reported that supplemental proteases derived from *Bacillus licheniformis* increased the relative abundances of beneficial bacteria, such as Bacteroides, *Lactobacillus*, *Alistipes*, and *Eubacterium*, in broilers. Therefore, supplemental various proteases appear to exert positive effects on the gut microbiota.

### 2.2. Effects of Dietary Protein Levels on the Prebiotic Roles of Supplemental Protease

Yang et al. [11] demonstrated that supplemental AP increased the abundance of beneficial gut bacteria in rats, whereas supplementation with heat-inactivated AP did not. This suggests that protease activity plays a significant role in the bifidogenic effect of AP. The researchers hypothesized that some amount of AP might evade the stomach’s acidic environment and subsequently enhance protein breakdown in the intestines, increasing the availability of free amino acids that could promote *Bifidobacterium* and *Lactobacillus* growth. Accordingly, they postulated that supplemental AP elevates free amino acid and beneficial bacterium levels in the cecum of rats on an adequate-protein diet but not in those on a low-protein diet [11]. Supporting this hypothesis, they found that AP supplementation significantly increased free amino acid levels and probiotic abundance in the cecal contents of rats on an adequate-protein diet but not in those of rats on a low-protein diet. Additionally, changes in the levels of several free amino acids were individually associated with the abundance of the probiotics. Thus, an adequate-protein diet may be required to achieve the prebiotic-like effect of AP, which occurs through increased free amino acid levels from dietary protein hydrolysis.

### 2.3. Effects of Supplemental Protease on Free Amino Acids in the Gut

Yang et al. [11] reported that supplemental AP, when combined with an adequate-protein diet, increased the levels of several free amino acids, including Thr, Ala, Pro, Tau, Cys, and GABA (γ-aminobutyrate). These amino acids exert gut-protective effects by improving intestinal barrier function through antioxidant and anti-inflammatory mechanisms [11]. Additionally, Cys specifically promotes *Bifidobacterium bifidum PRL2010* growth [19]. The AP-induced elevation of cecal Cys levels may partially be related to the increased abundance of beneficial bacteria. Recently, in vitro studies have demonstrated that several protein hydrolysates and peptides stimulate the growth of *Bifidobacterium* and *Lactobacillus* in comparison with undigested proteins [20]. The evidence supports the possibility of the involvement of protein digestion by the proteases in enriching beneficial bacteria. However, the information on the effects of the hydrolysates and peptides on the growth of other various bacteria including harmful bacteria is limited. Hence, it is unclear why dietary proteases specifically increase beneficial bacteria, but not other bacteria.

### 2.4. Other Effects of the Supplemental Proteases

As shown in Table 1, various protease supplements exert antioxidant and anti-inflammatory effects and enhance intestinal growth (villus height), morphology, intestinal barrier function, and immune function [5,14,15,17]. Importantly, these effects are comparable to those of probiotics, such as *Lactobacillus* and *Bifidobacterium*, and prebiotics, such as FOS and inulin [18]. Recent findings suggest that probiotics mitigate oxidative stress in both their own cells and the host’s through several mechanisms, including scavenging reactive oxygen species, chelating metal ions, increasing antioxidant enzyme levels, synthesizing non-enzymatic antioxidants, and producing metabolites with antioxidant properties [21]. Investigating whether exogenous digestive enzymes employ similar mechanisms to exert antioxidant effects would be of great interest.

## 3. Lipases

### 3.1. Effects of Supplemental Lipases on the Gut Microbiota

All four studies [6,13,22,23] listed in Table 2 demonstrated improved gut microbiota composition in animals receiving microbially derived supplemental lipases. Menden et al. [6] found that exogenous CL positively affected the fecal abundances of Christensenellaceae and *A. muciniphila* in wild-type mice, which was associated with improved health. Yang et al. reported that supplemental *Aspergillus* lipase (AL) and *Penicillium* lipase (PL) increased the relative abundances of *Bifidobacterium* and *Lactobacillus* in rats fed a high-fat diet [13,23]. Both lipases exhibited similar in vitro activities (60,000 and 55,000 U/g, respectively), but AL had a considerably higher prebiotic activity than did PL. In fact, supplementation with 0.1% AL induced a 120-fold increase in the relative abundance of cecal *Bifidobacterium*, whereas supplementation with 0.4% PL induced a 127-fold increase. AL is a typical acid lipase that remains stable under acidic conditions, whereas PL is easily inactivated in an acidic environment. Therefore, the acid-resistant property of AL may protect it from inactivation by stomach acid, allowing the active lipase to enter the colonic lumen, where it may partly favor the growth of beneficial bacteria by enhancing the utilization of triglycerides. A more acid-resistant lipase could have a more substantial prebiotic impact on the gut microbiota by enhancing their use of energy substrates. Therefore, exogenous acid lipases, along with the previously mentioned acid protease [11], may improve intestinal microbiota composition and health. However, the mechanism through which exogenous AL increases the abundance of probiotic bacteria in the gut is currently unknown.

### 3.2. Other Effects of the Supplemental Lipases

Menden et al. [22] showed that administering CL to APP/PS1 mice (Alzheimer’s disease mouse model) not only increased the fecal abundances of beneficial microbiota, such as *Acetatifactor* and Clostridiales, but also reduced the Alzheimer’s disease-like pathology. Furthermore, they demonstrated that the memory benefits produced by this approach could be transferred to antibiotic-induced microbiome-depleted wild-type mice with memory deficits via fecal microbiota transplantation. The Kyoto Encyclopedia of Genes and Genomes (KEGG) analysis by Yang et al. [23] showed that supplemental PL reduced the abundances of bacterial genes involved in the biosynthesis of lipopolysaccharide (LPS, a pro-inflammatory factor) and increased the abundances of those involved in the biosynthesis of D-amino acids (anti-inflammatory metabolites) in rats.

## 4. Overall Discussion

Consistent with our hypothesis (Figure 1), growing evidence suggests that exogenous proteases and lipases have a positive impact on gut microbial composition (Table 1 and Table 2). These beneficial effects occur regardless of the digestive enzymes originating from microorganisms or plants. Exogenous digestive enzymes facilitate the breakdown of food components into simpler forms to enhance nutrient digestion. Gut digesta may be a key factor in the growth of beneficial bacteria. Additionally, studies have shown that animals fed digestive enzymes have elevated levels of gut SCFAs, especially lactate [5,10,12,23], which acts as an anti-inflammatory mediator. Based on the available evidence, we propose that exogenous digestive enzymes may represent a novel category of prebiotics. Specifically, acid-resistant digestive enzymes, such as *A. oryzae*-derived acid protease and *A. niger*-derived lipase, which are resistant to inactivation in the acidic stomach environment, could have a greater positive effect on gut microbiota composition. Moreover, growing evidence suggests that exogenous proteases and lipases benefit other aspects of health, including inflammation, oxidative stress, intestinal morphology, and intestinal barrier function. These beneficial effects can be attributed to improvements in gut microbiota composition.

Notably, supplemental AP increased the gastrointestinal levels of free amino acids and the abundance of *Bifidobacterium* and *Lactobacillus* in rats fed an adequate-protein diet but not in those on a protein-deficient diet [11]. The enrichment of these bacteria was associated with increased levels of beneficial amino acids, such as Thr, Cys, Tau, GABA, Pro, and Ala [11]. Therefore, the beneficial effects of AP may be partly attributable to enhanced dietary protein digestion and the resulting elevation of free amino acids and peptides. Further in vitro studies are needed to assess how directly adding digestive enzymes to the gut digesta affects microbiota composition.

Fermented foods contain active digestive enzymes such as proteases and lipases, as do raw foods, such as uncooked fish, vegetables, and fruits. Given the beneficial effects of exogenous digestive enzymes, unheated fermented foods and raw foods likely support gut health through their digestive enzymes. Commercial digestive enzyme supplements have recently emerged for both animal and human use [3,4]. These supplements may help boost beneficial gut bacteria, which support gut health by modulating oxidative stress, inflammation, and intestinal barrier function. These digestive enzyme supplements may provide an important approach for boosting beneficial gut bacteria, which exert beneficial effects on gut health by modulating gut oxidative stress, inflammation, and barrier function. The overall evidence in this review suggests that the use of exogenous digestive enzymes promotes gut health by improving gut microbiota composition and increasing SCFA levels. To examine this possibility, further studies are necessary to explore the causal relationship between improved nutrient digestion and the gut microbiota in animals fed digestive enzymes. Digestive enzyme supplements can provide a significant strategy for promoting gut health by exerting prebiotic effects beyond food digestion. Furthermore, the discovery of the prebiotic effects of exogenous digestive enzymes has prompted the development of a new approach to producing a range of prebiotic foods through fermentation with fungi such as *Aspergillus*, producing large amounts of digestive enzymes.

Pancreatitis results in a deficiency of endogenous pancreatic digestive enzymes (exocrine pancreatic insufficiency: EPI), such as protease, lipase, and α-amylase, leading to impaired food digestion and dysbiosis [24,25]. Pancreatic enzyme replacement therapy using exogenous pancreatic enzyme preparations (generically called pancreatin) containing digestive enzymes is used to treat pancreatic diseases. Notably, recent studies have indicated that supplemental pancreatin increased gut *Lactobacillus* levels in mice [26,27]. These effects of pancreatin on the gut microbiota are comparable to those of supplemental proteases and lipases as mentioned above. Li et al. [28] demonstrated that *Bifidobacterium* and its metabolite, lactate, can protect pancreatitis in germ-free and antibiotic-treated mouse models. Thus, supplemental protease and lipase might be beneficial for patients with EPI by improving gut microbiota composition (Figure 1).

Conversely, protease expression is frequently elevated in gastrointestinal inflammatory diseases such as inflammatory bowel disease (IBD) [29]. Several studies on IBD have highlighted an imbalance between proteases and their inhibitors, of both host and bacterial origin [30]. Thus, clarifying the roles of exogenous digestive enzymes in the progression of inflammatory diseases with increased protease expression is essential. Although the apparent adverse effects of supplemental digestive enzymes have never been indicated in the animal studies listed in the tables, further studies on the safety, optimal dosage, and potential contraindications of digestive enzymes are necessary.

This review has some limitations. The studies cited in this review were conducted on healthy animals. Significant differences in gastrointestinal function, morphology, and microbiome composition exist between animals and humans. Therefore, the conclusions of these studies should be validated in humans before using digestive enzymes to modulate the human gut microbiota. Furthermore, research on the effects of supplemental digestive enzymes in animal models of inflammatory diseases—such as IBD, EPI [24], and Alzheimer’s disease [22]—is limited, highlighting the need for further investigation.

## 5. Conclusions

This is the first review of studies investigating our hypothesis that supplemental proteases and lipases act as prebiotics, benefiting the gut microbiota and overall health. Consistent with this hypothesis, a growing body of research shows that supplementation with various proteases and lipases yields benefits, including improved gut microbiota composition, increased production of microbial metabolites such as SCFAs, and reduced gut inflammation. Additionally, these digestive enzymes can improve intestinal morphology and barrier function in animals, possibly by promoting beneficial bacteria. In the case of exogenous proteases, their ability to enrich gut-protective amino acids may contribute to host health and disease prevention. Accordingly, digestive enzymes may have crucial roles in not only nutrient digestion, but also improvements in gut microbiota composition. Since fermented and raw foods often contain active digestive enzymes, their consumption may be favorable for gut health by enhancing the growth of beneficial bacteria. Further research is needed to investigate the roles of exogenous digestive enzymes in animal disease models and humans.

## Figures and Tables

**Table 1:** Effects of supplemental proteases on gut microbiota composition and related parameters in animals.

Citation	Animals	Study Design	Main Results
Yang et al. (2015) [5]	growing rats	Rats were fed a high-fat diet with or without 0.2% protease preparations (*n* = 9 per group), including Amano protease (AP: proteases from *A. oryzae* [Protease A “Amano” SD, protease activity at pH 6.0, 100,000 U/g] Amano Enzyme Inc.) or Orientase AY (OR: protease from *A. niger* [protease activity at pH 4.0; 200,000 U/g], HBI Enzymes Inc., Shiso, Japan) for three weeks.	Cecal *Bifidobacterium* was significantly elevated in the AP group but not in the OR group (1-way ANOVA and Tukey’s post hoc test). These proteases both increased cecal *Lactobacillus* and SCFA levels and fecal IgA and mucins. Cecal NH_3_ levels were unaffected.
Yang et al. (2017) [10]	growing rats	Rats were fed a high-fat diet containing 0.0384% purified acid protease (AcP: obtained from *A. oryzae*, Amano Enzyme Inc., Nagoya, Japan) or 0.1% AP for two weeks (*n* = 6 per group). Alkaline protease and amylase purified from *A. oryzae* were also tested to affect gut *Bifidobacterium* numbers.	Supplemental AcP significantly increased the cecal and fecal numbers of *Bifidobacterium* and cecal SCFA, especially lactate (1-way ANOVA and Tukey’s test). However, the supplementation of alkaline protease and α-amylase had no effect.
Yang et al. (2019) [11]	growing rats	Rats were fed a 0.1% AP-supplemented or -unsupplemented diet with a low protein (3% casein) or adequate-protein (25% casein) diet for two weeks (*n* = 8 per group).	Dietary AP significantly increased cecal *Bifidobacterium* and *Lactobacillus* and the levels of beneficial amino acids such as Thr, Ala, Cys, Tau, and GABA in the rats fed with an adequate-protein diet, but not in those fed with a low-protein diet (2-way ANOVA and Tukey’s test). Cecal NH_3_ levels were unaffected by AP.
Yang et al. (2021) [12]	growing rats	Rats were fed the high-fat diet supplemented with or without AP or AL (*A. niger*-derived lipase preparation, Amano Enzyme Inc., Nagoya, Japan) at 0.1% for two weeks (*n* = 6 per group).	AP significantly elevated the cecal relative abundances of *Bifidobacterium*, *Collinsella*, and *Enterococcus*, but reduced those of harmful *Oscillospira*, *Dorea*, and *Coprobacillus* (1-way ANOVA and Tukey’s test). Additionally, AP increased cecal lactate levels.
Kostiuchenko et al. (2022) [13]	growing mice	In experiment 1, mice were fed with or without bromelain. In experiment 2, mice were fed with or without papain. The proteases were obtained from Taconic Biosciences Inc., Seattle, WA, USA. The proteases were administered in a dose of 1 mg per animal/day for three days (*n* = 6 or 7 per group).	Fruit proteases significantly decreased the fecal abundance of pathogenic proteobacteria and increased beneficial *A. muciniphila* (Student’s *t*-test).
Peng et al. (2022) [14]	weaned pigs	Pigs were fed on four types of diets (control diet (CON), CON + protease, CON + essential oil (EO), and CON + protease + EO (*n* = 8 per group) for 14 days. Protease was supplemented at 500 mg/kg protease (alkaline serine endopeptidase derived from *B. licheniformis*, protease activity of 600,000 units/g. Cibenza DP100, Novus International, Inc., St. Louis, Missouri, MO, USA).	The major effects of protease were to significantly improve intestinal morphology (ratio of villus height to crypt depth) and to increase the colonic abundance of probiotic Actinobacteria (2-way ANOVA and Duncan’s multiple-range test). Additionally, protease reduced serum markers of lipid peroxidation and oxidative stress.
Peng et al. (2024) [15]	growing pigs	Pigs were fed on three types of sorghum-based diets ((1) control diet (CON), (2) CON + 200 mg/kg proteases, and (3) CON + 400 mg/kg proteases) for 21 days (*n* = 8 per group). The proteases (KEMZYMETM, Kemin Industries, Des Moines, IA, USA) consist of alkaline protease from *Bacillus Licheniformis* (6850 units/g), neutral protease from *Bacillus subtilis* (8120 units/g), and acidic protease from *Aspergillus niger* (1700 units/g).	Dietary 400 mg/kg proteases significantly improved the growth and health status of pigs (1-way ANOVA and Duncan’s multiple-range test) relating to blood parameters for protein metabolism and immunity, associated with the altered fecal microbiota composition, particularly the enrichment of SCFA-producing bacteria (Prevotella_9).
Yi et al. (2024) [16]	broilers	Chickens were fed the diet supplemented with alkaline protease derived from *B. licheniformis* (Bestzyme ProMax, Bestzyme Bio-Engineering Co., Nanjing, China) at doses of 0, 100, 200, 300, and 400 mg/kg for 42 days (*n* = 8 per group).	Higher levels of protease significantly increased the cecal relative abundances of beneficial *Bacteroides*, *Lactobacillus*, *Alistipes*, and *Eubacterium* (1-way ANOVA and Tukey’s test).
Liu et al. (2024) [17]	weaned piglets	Ninety weaned pigs were fed three types of diets for 28 days: (1) control, a basic diet with composite enzymes without protease; (2) negative control, a diet with no enzymes; and (3) dietary protease (PR), a control diet with protease. The proteases were composed of alkaline protease from *B. licheniformis* (6850 units/g), neutral protease from *B. subtilis* (8120 units/g), and acidic protease from *A. niger* (1700 units/g) (Kemin Industries, Des Moines, IA, USA) (*n* = 5 or 6 per group).	Dietary proteases significantly enhanced growth and increased the antioxidant capacity (1-way ANOVA and Tukey’s test). Protease addition reduced inflammatory markers. Protease supplementation significantly improved intestinal morphology and barrier integrity. Protease addition significantly increased the abundance of beneficial bacteria (*Lachnospiraceae_AC2044_group* and *Prevotellaceae_UCG-001*) and reduced harmful *Terrisporobacter*.

**Table 2:** Effects of supplemental lipases on gut microbiota composition and related parameters in animals.

Citation	Animals	Study Design	Key Results
Menden et al. (2020) [6]	growing mice	Mice received 1000 *Fédération Internationale Pharmaceutique* (FIP)/kg *C. rugosa* lipase (CL, Enzymedica, Venice, FL, USA) per day in drinking water for 30 days (*n* = 10 per group).	The fecal abundances of *Christensenellaceae* and *A. muciniphila* were significantly higher in the lipase-treated animals (ANCOM analysis).
Yang et al. (2021) [13]	growing rats	Rats were fed diets supplemented with *A. niger*-derived lipase preparation (AL, Lipase AP12 [lipase activity at pH 6.0, 60,000 U/g]. Amano Enzyme Inc. Nagoya, Japan) or AP at 0.1% for two weeks (*n* = 8 per group).	Dietary AL significantly elevated the cecal relative abundances of *Bifidobacterium*, *Collinsella*, and *Enterococcus*, but significantly reduced those of *Oscillospira*, *Dorea*, and *Coprobacillus* (1-way ANOVA and Tukey’s test). AL increased cecal SCFA, especially lactate.
Menden et al. (2022) [22]	growing APP/PS1 mice	APP/PS1 mice (Alzheimer’s disease mouse model) received 5000 FIP/kg body weight CL in drinking water for two months (*n* = 6 per group).	CL administration significantly reduced Alzheimer’s disease-like pathology by increased lipid hydrolysis in the gut lumen, which significantly increased the fecal abundances of *Acetatifactor* and *Clostridiales vadin BB60* genera and altered the gut metabolome and rebalanced energy metabolism (ANCOM analysis).
Yang et al. (2023) [23]	growing rats	Rats were fed diets supplemented with either *P. camemberti*-derived lipase preparation (PL, Lipase G “Amano” 50 [lipase activity of PL at pH 6.0, 55,000 U/g], Amano Enzyme Inc., Nagoya, Japan) at 0%, 0.2% or 0.4% for two weeks (*n* = 6 per group).	Dietary PL significantly increased the cecal relative abundance of beneficial bacteria such as *Bifidobacterium*, *Lactobacillus*, and *Collinsella* (1-way ANOVA and Tukey’s test). Dietary PL significantly modulated the relative abundances of bacterial genes associated with the biosynthesis of LPS and D-amino acids. Dietary PL increased cecal lactate.

## Abbreviations

The following abbreviations are used in this manuscript:

AP	Amano protease
OR	Orientase AY
SCFA	short-chain fatty acid
AcP	*Aspergillus* acid protease
Thr	threonine
Ala	alanine
Pro	proline
Tau	taurine
Cys	cystine
GABA	γ-aminobutyrate
FOS	fructo-oligosaccharide
CON	control
EO	essential oil
CL	*Candida rugosa* lipase
AL	*Aspergillus* lipase
PL	*Penicillium* lipase
KEGG	Kyoto Encyclopedia of Genes and Genomes
LPS	lipopolysaccharide
EPI	exocrine pancreatic insufficiency
IBD	inflammatory bowel disease
